# Tissue-Specific Effects of Aging on Repeat-Mediated Mutation Hotspots In Vivo

**DOI:** 10.3390/biom14111453

**Published:** 2024-11-16

**Authors:** Alexandra M. D’Amico, Tonia T. Li, Karen M. Vasquez

**Affiliations:** Division of Pharmacology and Toxicology, Dell Pediatric Research Institute, College of Pharmacy, The University of Texas at Austin, 1400 Barbara Jordan Blvd., Austin, TX 78723, USA; amdamico@utexas.edu (A.M.D.); tonia.li@utexas.edu (T.T.L.)

**Keywords:** aging, genetic instability, cancer, triplex DNA, DNA repair

## Abstract

Aging constitutes complex and dynamic alterations in molecular and physiological processes and is associated with numerous disorders, in part due to increased genetic instability. The aging population is projected to double by 2050, underscoring the urgent need to better understand the relationships between aging and age-related disorders. Repetitive DNA elements are intrinsic sources of genetic instability and have been found to co-localize with mutation hotspots in human cancer genomes. In this study, we explored the relationship between aging and DNA repeat-mediated genetic instability in vivo using an H-DNA-forming mirror-repeat sequence from the cancer-associated human *c-MYC* gene. Utilizing a unique mutation-reporter mouse model, we observed tissue-specific effects of aging on H-DNA-induced genetic instability, with mutation frequencies increasing in spleen tissues and remaining unchanged in testis tissues. Analysis of the mutation spectra revealed large deletion mutations as the primary contributor to increasing H-DNA-induced mutations, supported by increased cleavage activity of H-DNA structures in aged spleen tissues. Our findings demonstrate that aging has distinct tissue-specific effects on repeat-mediated, cancer-associated mutations, providing insights into the complex relationship between aging and cancer.

## 1. Introduction

With the global aging population booming in recent years and increased projections for future decades, it is imperative that we understand the increased risks of diseases that are associated with aging and their impact on public health. Several diseases are linked to increasing age, including cancer, diabetes, cardiovascular disease, arthritis, and neurological disorders. Cancer remains primarily a disease of aging, with half of new cancer cases diagnosed amongst the 65-year-and-older population [1].

A hallmark common to both cancer and aging is a loss in genomic integrity [2,3]. Genetic instability has long been established as a consequence of aging and an initiator of cancer. Mutation hotspots in the genome are commonly found in regions of repetitive DNA elements, which have the capacity to adopt alternative DNA structures (i.e., non-B-DNA) that differ from the canonical B-DNA structure described by Watson and Crick [4]. Several types of repeat-mediated DNA structures have been identified, including H-DNA, which forms at mirror-repeat sequences in the genome [5,6]. Named for its requirement of H^+^ in the Hoogsteen hydrogen bonds holding the intramolecular triplex structure together, H-DNA-forming sequences are intrinsically mutagenic and enriched at translocation breakpoint hotspots in human cancer genomes, including leukemias and lymphomas [7,8].

H-DNA structure formation, like other non-B repeat-mediated DNA structures, is stabilized by negative supercoiling generated during DNA replication, transcription, and repair [9]. Once formed, H-DNA has been shown to cause replication fork stalling, impede transcription complexes, and can be recognized and processed by DNA repair and replication proteins in both error-free and error-prone manners [9,10]. For example, H-DNA has been shown to stimulate the formation of DNA double-strand breaks (DSBs) during its processing, which can result in large deletions via error-generating repair mechanisms [5,10,11].

Despite being a common hallmark to aging and cancer, the influence of age on repeat-mediated genome instability at cancer-associated mutation hotspots is poorly understood. Here, we sought to provide insights into this relationship using an engineered mutation-reporter mouse model we previously constructed to contain either a control B-DNA-forming sequence or a mirror-repeat H-DNA-forming sequence from the human cancer-associated *c-MYC* oncogene [11,12,13]. Using this model, we previously demonstrated that H-DNA-forming sequences resulted in large deletion mutations and genetic instability in vivo using PCR-based mutation analysis [11]. More recently, our lab has shown that diet-induced obesity in these mice resulted in increased H-DNA-induced mutagenesis in multiple tissues [12]. In this study, our aim was to discern the impact of another factor of genetic instability, aging, on H-DNA-induced mutagenesis. Intriguingly, we observed tissue-specific effects of aging on H-DNA-induced genomic instability. Further analysis into the mutation spectra revealed striking age- and tissue-specific alterations in the H-DNA mutation spectra profile, supported by distinct modulation of H-DNA cleavage activity in tissue-derived extracts.

## 2. Materials and Methods

### 2.1. Mice

The genetically engineered mutation-reporter mice in this study were established as previously described [11]. FVB/N male mice with chromosomally integrated mutation reporters containing either a human B-DNA sequence or an H-DNA mirror-repeat sequence from the human *c-MYC* gene were re-derived from frozen embryos and aged to 2- and 18-months of age (N ≥ 3 per age group per genotype) [11,12]. Spleen and testis tissues were harvested at each age endpoint and stored at −80 °C until ready for processing. Mice were housed and euthanized in accordance with IACUC guidelines.

### 2.2. Genomic DNA Extraction and Rescue of the LacI/LacZ Mutation Reporter

**Figure 1:**
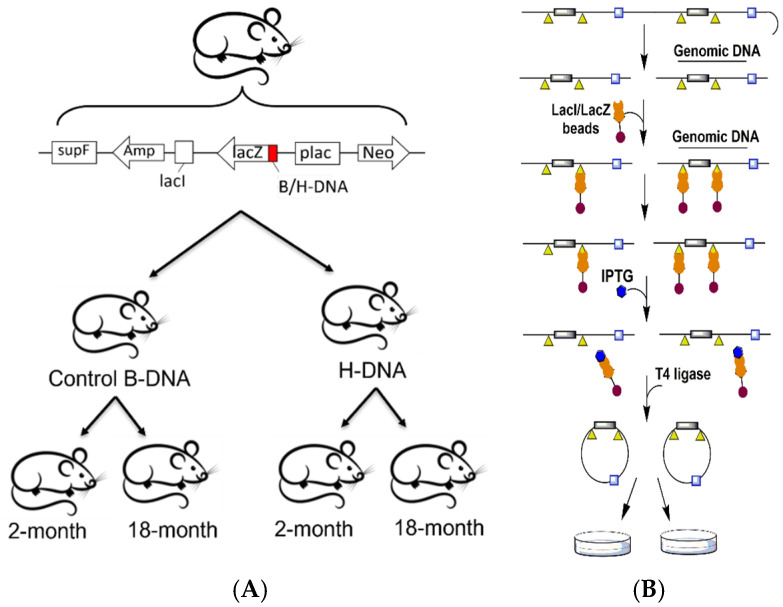
Schematic of p2RT mutation reporter rescue from the genomic DNA of male spleen and testis tissues. (**A**) Male mice (N ≥ 3) containing a genomically integrated mutation reporter with an H-DNA-forming or B-DNA-forming sequence were aged to 2- and 18-months of age. (**B**) The integrated mutation reporters were recovered from the mouse genome by restriction enzyme digestion followed by magnetic bead rescue using lacI-lacZ-conjugated magnetic beads. The isolated mutation reporters were purified, re-circularized, and electroporated into DH10β cells for blue–white screening. A minimum of 15,000 colonies per mouse tissue in each age group were counted for mutation frequency analysis. A random selection of white mutant colonies were sequenced for determination of mutation spectra. Gray rectangles, *lacZ* mutation-reporter gene; yellow triangle, *lacI* binding site.

Genomic DNA from mouse spleen and testis tissues were extracted by mincing tissues with a sterile blade and overnight incubation at 50 °C in DNA lysis buffer (0.5 mM EDTA, 20 mM Tris-HCl pH 8.0, 400 mM NaCl, 1% SDS, and 1 mg/mL proteinase K). DNA was purified by phenol:chloroform extraction and ethanol precipitation. To recover the chromosomally-integrated mutation reporter from the genomic DNA, the mutation reporter contains two *spe1* restriction sites. Upon treatment with the spe1 restriction enzyme (NEB, Ipswich, MA, USA), the reporter is released from the genomic DNA and available for magnetic bead rescue and purification. Approximately 50 μg of genomic DNA was treated with spe1 restriction enzyme followed by incubation with magnetic beads conjugated to a lacI/lacZ fusion protein, which bound to the *lacI* binding sites on the reporter. The mutation reporter was recovered by IPTG elution, re-circularized with DNA ligase, and purified by ethanol precipitation (Figure 1B) [11,14,15].

### 2.3. Mutation Screening

The recovered mutation reporter was electroporated into DH10B bacterial indicator cells (Invitrogen, Waltham, MA, USA) and plated on agar plates containing carbenicillin, IPTG, and X-gal, and analyzed via blue–white screening for determination of mutation frequencies and spectra. Mutations within and/or surrounding the H-DNA or control B-DNA sequences resulted in non-functional *lacZ* genes and yielded white bacterial colonies. Functional *lacZ* genes (i.e., no mutation) yielded wild-type blue colonies. A minimum of 15,000 colonies were counted per mouse tissue sample per age group for robust determination of mutation frequencies, calculated as the number of white (mutant) colonies divided by the total number of colonies (blue + white). The mutation reporter was purified from a random sample of mutants and sent for sanger sequencing at our core facility for mutation spectra analysis. Sequences were aligned to reference sequences using NCBI BLAST and mutations characterized into large deletions (>30 bp) and point mutations. The point mutation category includes base substitutions, insertions, and small deletions, the majority of which were single base pair mutations.

### 2.4. H-DNA Cleavage Assay

Whole-cell extracts were prepared by crushing tissues under liquid nitrogen into a fine powder and treating with mild protein lysis buffer [50 mM Tris-HCl pH 8.0, 150 mM NaCl, 1 mM EDTA, 0.1 mM DTT, 1% NP-40, supplemented with a protease and phosphatase inhibitor cocktail (Roche, Basal, Switzerland)] at 4 °C for 1–2 h. Debris was removed by centrifugation and protein concentration determined using the Pierce BCA assay (ThermoFisher Scientific, Waltham, MA, USA). For the in vitro cleavage assay, 6 nM of R25′ oligo was annealed as previously described and incubated with 50 μg of 2-month or 18-month whole-cell extracts in reaction buffer (40 mM Tris-HCl pH 7.5, 10 μM MgCl_2_, 5 mM DTT, and 200 μg/mL BSA) for 10 min at 30 °C [10]. Reactions were stopped with EDTA and proteins removed by proteinase K and SDS treatment. DNA was purified by ethanol precipitation and separated on a 12% denaturing urea-polyacrylamide gel. The gels were exposed to phosphor screens, imaged, and quantified using the Typhoon Imaging System (GE Healthcare, Marlborough, MA, USA) and ImageQuant TL7 Analysis software. Cleavage products were presented as a ratio of cleaved oligonucleotide products relative to unprocessed full-length oligonucleotide substrate and compared across age groups.

### 2.5. Statistical Analysis

Statistical analysis was performed using GraphPad Prism v9 software. Each age group had a minimum N ≥ 3 biological replicates for scientific rigor and reproducibility. Significant changes in mutation frequency and spectra data were determined using one-way ANOVA followed by Šidák’s post hoc analysis. Cleavage products were analyzed and tested for significance using an unpaired Student’s *t*-test.

## 3. Results

### 3.1. Aging Demonstrates Tissue-Specific Effects at H-DNA Mutation Hotspots In Vivo

**Figure 2:**
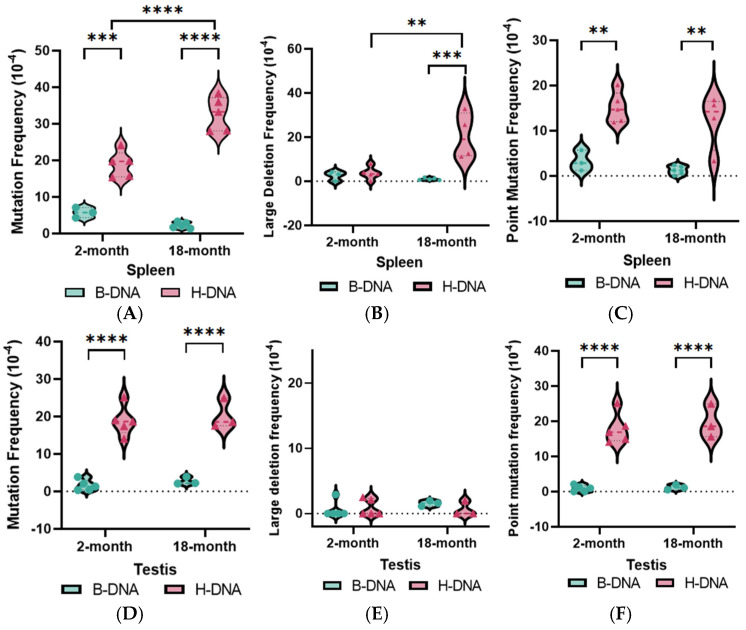
H-DNA-induced genetic instability increased with age in spleen tissues but not testis. Mutation frequencies of (**A**) spleen and (**B**) testis tissues from 2- and 18-month H-DNA and B-DNA mice. Mutants from the blue–white screening assay were characterized by DNA sequencing and plotted as the respective portion of the overall mutation frequency. Large deletion frequencies from (**C**) spleen and (**D**) testis tissues and point mutation frequencies from (**E**) spleen and (**F**) testis tissues from 2- and 18-month H-DNA and B-DNA mice. Data from biological replicates (N ≥ 3) are plotted as violin plots with individual data points (circles, 2-months; triangles, 18-months) and dotted lines indicating median. Statistical analyses were performed using one-way ANOVA followed by Šidák’s post hoc test. ** *p* < 0.01, *** *p* < 0.001, **** *p* < 0.0001.

To determine the impact of age on H-DNA-induced genetic instability, transgenic mice carrying a chromosomally integrated mutation-reporter vector, containing either an H-DNA-forming sequence from the human *c-MYC* gene or a B-DNA-forming sequence, were aged to 2- and 18-months of age (Figure 1A). The H-DNA- or control B-DNA-forming sequence is located within a *lacZ* mutation-reporter gene, allowing for downstream blue–white screening analysis of the mutations that occurred in the mouse genome. The mutation reporter was recovered from the genomic DNA of spleen and testis tissues using lacI/lacZ magnetic bead rescue and screened for changes in mutation frequencies and spectra via blue–white screening analysis (Figure 1B). In both spleen and liver tissues, H-DNA demonstrated significant mutagenic effects compared to B-DNA in the 2-month age group and 18-month age group. Interestingly, we observed age-associated tissue-specific patterns of genetic instability in the H-DNA mice, with a significant increase in H-DNA-induced mutations in spleen tissues, while testis tissues demonstrated no significant differences (Figure 2A,B).

### 3.2. H-DNA Mutation Spectra Profiles Are Age- and Tissue-Specific

A random sample of the mutants from the blue–white screening assay were characterized by DNA sequencing (Tables S1 and S2). In-depth analysis of the mutations induced in the mice revealed a significant increase in the frequency of H-DNA-induced large deletions in spleen tissues with age (Figure 2C, Table S1). This increase is consistent with the increase in overall mutagenesis observed in Figure 2A, suggesting that the increase in H-DNA-induced mutagenesis in the spleen may be primarily due to an increase in H-DNA-induced large deletion mutations. In contrast, there were no significant changes in the H-DNA mutation spectra profile in testis tissues with age (Figure 2D, Table S2), consistent with the lack of change in the overall H-DNA-induced mutagenesis observed in Figure 2B). Additionally, the predominant mutation type caused by H-DNA in spleen tissues appeared to change from point mutations at 2-months of age to large deletions at 18-months of age (Figure 2E). This mutation type switching appeared to be unique to the spleen as point mutations remained the predominant H-DNA mutation type in testis tissues across the two age groups (Figure 2F).

### 3.3. Cleavage of the H-DNA Structure Is Increased with Age in Spleen Tissues

**Figure 3:**
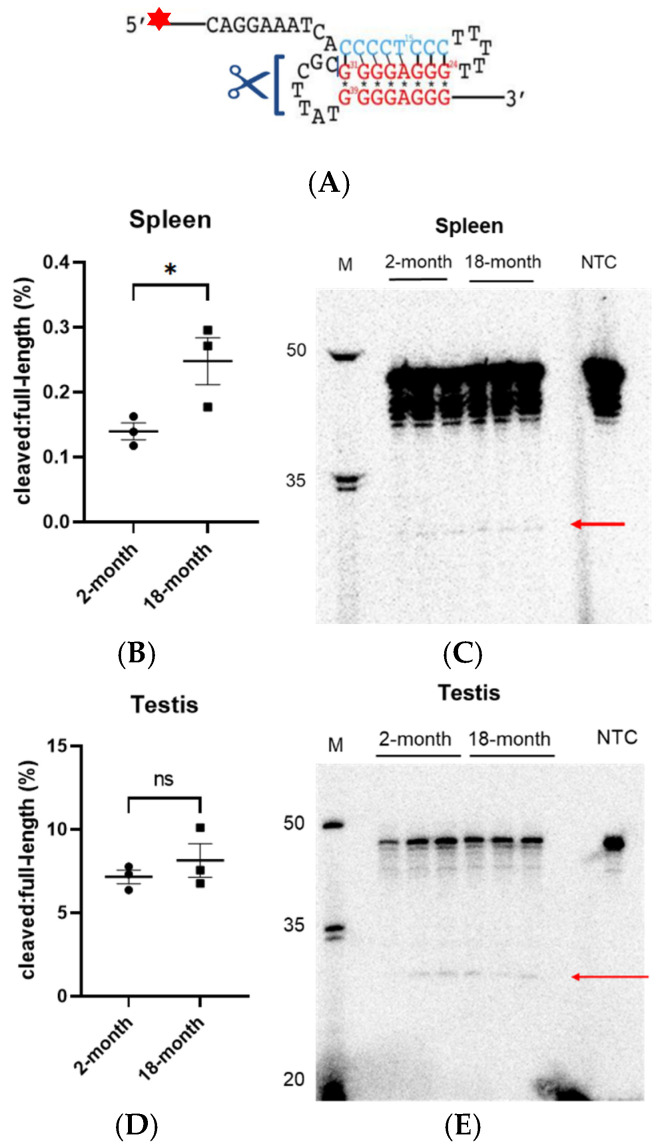
Aging leads to an increase in cleavage activity on an H-DNA-forming structure in spleen whole-cell extracts. (**A**) A 5′-labeled H-DNA structure (R25′, adapted from Del Mundo et al., 2017 [18]) was treated with whole-cell extracts derived from 2- and 18-month spleen and testis tissues (N = 3) and separated by denaturing urea-PAGE. Each lane contains recovered R25′ full-length substrate and cleavage products from a mouse tissue extract (N = 1 mouse extract per lane). (**B**) Spleen and (**C**) testis gels were imaged using Typhoon imaging system. Quantification of cleavage products generated relative to full-length oligo from (**D**) spleen and (**E**) testis extracts was performed using ImageQuant TL 7 analysis software. Cleavage products are indicated by red arrows. M, DNA ladder. NTC, non-treated control. Individual data points are plotted as black squares. Error bars represent mean ± SEM. Statistical significance determined by Student’s *t*-test. * *p* < 0.05, ns = not significant. Original images can be found in Supplementary Materials.

The non-canonical structure of H-DNA can result in error-prone processing by cellular machinery, and as such, H-DNA is inherently mutagenic [5,10,16]. Additionally, with the decreased fidelity of DNA processing with age the potential for erroneous DNA cleavage events becomes more likely [17]. Here, we set out to determine how aging affects the processing of a cancer-associated H-DNA-forming sequence using whole-cell extracts derived from spleen and testis tissues of 2- and 18-month-old male mice. We have previously constructed and characterized an oligonucleotide-based H-DNA-forming substrate, R25′, and found that it is recognized and cleaved by multiple H-DNA-related repair and replication proteins, making it a useful tool for biochemical analysis of H-DNA processing events [10,18]. Indeed, subjecting the H-DNA-forming oligo to incubation in tissue-derived whole-cell extracts from 2- and 18-month aged mice revealed a unique 31-nucleotide cleavage product that was present in both spleen and testis (Figure 3A). The size of this product suggested that the cleavage event occurred on the DNA loop between the duplex and third triplex strand.

Due to the excessive nuclease activity of the spleen, we observed significant DNA degradation in cleavage products recovered from spleen extracts that made visible differences challenging to discern. However, precise digital quantification using the Typhoon Imaging system and ImageQuant revealed a modest but significant increase in the cleavage of the H-DNA substrate in 18-month spleen whole-cell extracts relative to the unprocessed full-length substrate, consistent with the increase in H-DNA-induced large deletions with age (Figure 3B,C). In contrast, testis-derived whole-cell extracts demonstrated no differences in cleavage activity with increasing age, suggesting that the processing of the H-DNA structure, at least in mouse tissue extracts, remains unchanged in testis tissues (Figure 3D,E).

## 4. Discussion

In this study, we provide critical insights into the relationship between aging and a cancer-associated mutation hotspot in the context of repeat-mediated genetic instability. We observed distinct age-associated tissue-specific impacts on H-DNA-induced genetic instability, with mutation frequencies increasing in spleen tissues of H-DNA mice with age, while remaining unchanged in testis tissues. Analysis of the H-DNA mutations in spleen tissues revealed dynamic effects of age on the H-DNA-induced mutation spectra profile, with the predominant mutation type changing from point mutations to large deletions mutations with increasing age. Together, these data suggest that the increase in H-DNA-induced instability in spleen tissues with age is primarily due to an increase in H-DNA-induced large deletion mutations. In contrast, the point mutations remained the predominant H-DNA-induced mutation type in testis tissues. These results were intriguing, as previous in vitro studies have shown that that majority of H-DNA-induced mutations were large deletions in mammalian cells [5]. The observed tissue-specific differences in H-DNA mutation patterns are likely due to the increased complexity of our non-B-DNA mutation-reporter system being integrated into the mouse genome, providing a more relevant context to human health than cellular models. This finding also provides insight into a potential mechanism for the increase in *BCL-2* translocation events with age, a common mutation in follicular lymphoma and previously suggested to be the result of triplex DNA-induced genomic instability [19,20,21].

The increase in cleavage activity congruent with the rise in H-DNA mutagenesis suggests alterations in protein activity and/or efficiency in H-DNA processing. Studies have identified distinct, tissue-specific alterations in the proteasomes of mouse organs with age [22,23]. Further, protein homeostasis and quality control mechanisms, including recognition of misfolded proteins, have been reported to decline with age [24,25,26]. Several groups have also reported tissue-specific alterations in the expression of DNA repair proteins with age [27,28,29,30]. This may, at least partially, explain why altered cleavage activity appeared to be specific to aged spleen tissues. That cleavage activity increased with age in the spleen and not in the testis, along with only the spleen having increased H-DNA-induced mutagenesis with age, suggests that the increased cleavage likely contributed to the accumulation of H-DNA-induced large deletions.

The biological function of a tissue also plays a crucial role when considering the effects of age on genomic instability. As negative supercoiling is a catalyst for H-DNA formation, it is likely that tissues with high proliferation rates have a greater opportunity for H-DNA structure formation and genomic instability [16,18,31]. Spleen and testis tissues are both considered to be proliferative tissues for their respective biological functions. However, while H-DNA-induced mutagenesis was dynamic in the somatic spleen organ, the mutation frequencies and spectra were static with age in gonadal tissues. This lack of H-DNA-induced mutation accumulation is likely to preserve DNA integrity for genomic inheritance, a function unique to the testis tissues [32,33,34]. For example, several groups have found that testis tissues have robust DNA repair activity including end-joining repair, a critical repair pathway for DNA double-strand breaks that are induced by H-DNA [35,36,37,38]. V(D)J recombination events and declining DNA repair efficiencies with age in spleen tissues may contribute to the increase in H-DNA-induced large deletions mutations we observed [39,40]. This diversity in tissue processes further highlights the impact of canonical organ function and tissue-specificity when studying the relationships between age-related disorders and genome stability.

## 5. Concluding Remarks

The rise in age-associated cancer incidence prompts understanding of the relationship between aging and cancer initiation and development. Alternative DNA structures such as H-DNA are an intrinsic source of genomic instability and have been mapped to translocation breakpoint hotspots in human cancer genomes. We have previously established that H-DNA induces genetic instability in several models, including yeast, mammalian cells, and mice, and is regulated by structure-specific DNA repair processes [5,10,11,12]. In this study, we provide preliminary evidence that aging leads to unique, tissue-specific effects at cancer-associated H-DNA-forming sequences in vivo. Future studies investigating the effects of age on H-DNA mutagenesis in a broader range of tissues and specific cell types are necessary to further interrogate the mechanisms linking aging, DNA structure-induced instability, and cancer. Ultimately, these studies will provide insights into the mechanisms resulting in disease-associated genetic instability, allowing for the development and improvement of therapeutic strategies to treat and/or prevent age-associated diseases.

## 6. Limitations of This Study

One limitation of this study is the excessive nuclease activity present in cell-free tissue extracts, most notably spleen as seen in Figure 3C, making overall cleavage efficiency and DNA recovery challenging. Although a known challenge of DNA repair assays in tissue extracts, with the enhanced specificity and linear dynamic range of phosphor screens we were able to quantify the ratio (%) of cleavage products (red arrow) to full-length oligo (top band near 50 nt ladder marker) on the urea-polyacrylamide gel using the Typhoon Imaging System and ImageQuant TL 7 software analysis for radioactive gels. Even with the significant nuclease activity in the spleen yielding small cleavage ratios, we detected a statistically significant difference between products from young versus aged extracts.

## Data Availability

The data that support the findings of this study are available from the corresponding author upon reasonable request.

## Supplementary Materials

The following supporting information can be downloaded at: https://www.mdpi.com/article/10.3390/biom14111453/s1; Table S1: Mutation spectra distribution of mutants from 2-month and 18-month control B-DNA and H-DNA spleen tissues. Table S2: Mutation spectra distribution of mutants from 2-month and 18-month control B-DNA and H-DNA testis tissues. Original images can be found in Supplementary Materials.
