# Supplementary material for: Tissue-Specific Effects of Aging on Repeat-Mediated Mutation Hotspots In Vivo
Source: Biomolecules. 2024 Nov 16;14(11):1453. doi: 10.3390/biom14111453 (PMC11592361; doi:10.3390/biom14111453)
Supplement: Supplementary file 1 [file biomolecules-14-01453-s001.zip › biomolecules-3198362-supplementary.pdf]

Table S1. Mutation spectra distribution of mutants from 2-month and 18-month control B-DNA and H-DNA spleen tissues.

	Large Deletions		Small Deletions		Insertions		Base Substitutions	
	Type	Location (bp)	Type	Location (bp)	Type	Location (bp)	Type	Location (bp)
**2-month Control**	3000-3500		G	857, 884, 909, 977 (2)			G>A	1105
	3500-4000	from 935, from 888						
	>4000	to 4595 (3), to 5254, to 4553 (5)	C	937 (2)				
**18-month Control**	3000-3500	1243-4553 (3)	G	1193, 1198, 1200, 1215, 1325	C	629	G>A	1335
	3500-4000	from 943, 303-4118	C	971	T	603		
	>4000	320-4595 (4), 385-4553	A	975, 1322 (2), 1186				
			T	899 (2), 1028				
**2-month H-DNA**	3000-3500	712-4123, 944-4121, 855-4122	G	970, 976	A	955, 1115	G>C	640, 771 (2), 779, 824, 909
	3500-4000	to 4120, to 4152					G>T	654 (2), 771, 930 (2), 976
	>4000						C>T	849
							A>G	869
							G>A	922, 968
							A>C	979
							T>G	913
**18-month H-DNA**	3000-3500	963-4372	C	902	T	795, 899, 957	T>G	728, 819, 1205
	3500-4000	623-4570 (2), 756-4499, 421-4123 (3), 418-4118	A	975	G	1001	G>A	985, 1145
	>4000				A	958	G>T	968

Table S2. Mutation spectra distribution of mutants from 2-month and 18-month control B-DNA and H-DNA testis tissues.

	Large Deletions		Small Deletions		Insertions		Base Substitutions	
	Type	Location (bp)	Type	Location (bp)	Type	Location (bp)	Type	Location (bp)
**2-month Control**	3000-3500		G	884, 909, 954, 989	A	1196		
	3500-4000	from 254, from 585	AA	919-920				
	>4000	to 4554, to 4553 (3)						
**18-month Control**	3000-3500	1243-4553 (5)	T	959	A	1341 (3)	G>T	771
	3500-4000	333-4122, 320-4126 (4)	A	859, 1341			G>A	1340
	>4000	608-4624						
**2-month H-DNA**	3000-3500	1074-4121	G	958 (2), 904	G	955	G>A	922 (2), 968 (2), 873 (2)
	3500-4000	320-4126	A	1292			G>C	795 (2), 781 (3), 772, 968, 771 (6), 1000
	>4000						G>T	695 (5), 653, 781, 856
							A>G	892, 788
							T>G	896
**18-month H-DNA**	3000-3500	1018-4122	G	1323 (7), 1324, 1335 (3)	A	1337, 1299	G>T	844, 832, 697, 650, 700
	3500-4000		T	1308 (2)	G	1182	T>A	966, 954
	>4000		A	1342			G>C	968, 771, 650
							C>T	651
							G>A	700, 909
